# Trans-aortic left ventricular thrombo-embolectomy following COVID-19 infection

**DOI:** 10.1093/jscr/rjab426

**Published:** 2021-09-27

**Authors:** Michael Janula, Andre Navarro, John Bonello, Kevin Schembri, Alex Borg

**Affiliations:** Department of Cardiothoracic Surgery, Mater Dei Hospital, Msida, Malta; Department of Cardiothoracic Surgery, Mater Dei Hospital, Msida, Malta; Department of Cardiology, Mater Dei Hospital, Msida, Malta; Department of Cardiothoracic Surgery, Mater Dei Hospital, Msida, Malta; Department of Cardiology, Mater Dei Hospital, Msida, Malta

## Abstract

Left ventricular thrombosis is a known complication of myocardial infarction. COVID 19 has been shown to produce a procoagulant state resulting in venous and less commonly arterial thrombosis. Here, we describe a patient who presented with a non-ST elevation myocardial infarction (NSTEMI), in the context of a COVID 19 infection. This NSTEMI resulted in the formation of a large pedunculated apical thrombus, which was initially managed conservatively, however ultimately required surgical thromboembolectomy. Access to the left ventricle was gained via the transaortic route in order to avoid ventriculotomy in a patient with a reduced LV systolic function. Post-operative imaging confirmed complete resection of thrombus.

## INTRODUCTION

The SARS-CoV-2 virus pandemic has placed a severe logistic, economic and societal burden on the global health sector, particularly on acute services [1]. Although the predominant symptoms of COVID-19 are respiratory, there are less well-defined cardiovascular complications, including a hypercoagulabile state that may result in venous and arterial thromboembolism [2]. We report a COVID-19 patient who sustained an acute non-ST elevation myocardial infarction (NSTEMI), complicated by left ventricular (LV) thrombus formation, ultimately resulting in recurrent embolic strokes. After failure of initial conservative medical management, he required open surgery, with access to the LV gained by a transverse aortotomy.

## CASE PRESENTATION

A 47-year-old gentleman suffering from diabetes mellitus, obesity and dyslipidaemia presented to the emergency department with severe chest pain radiating to both upper limbs, preceded by a week’s history of fever. On admission, he was haemodynamically stable, ECG showed a right bundle branch block and serum biochemistry showed a raised troponin T level of 504 mg/ml. A CT pulmonary angiography showed no evidence of pulmonary embolism; however, the patient had pulmonary findings consistent with a COVID-19 pneumonia. Provisional diagnosis was a non-ST elevation myocardial infarction (NSTEMI) in the context of COVID-19 pneumonia. This was confirmed by a positive rt-PCR SARS-CoV-2 test.

The patient was started on dual antiplatelet therapy and anti-coagulated using low molecular weight heparin (LMWH). A bedside echo showed impaired LV ejection fraction of 35%, with akinesia at the apex, anteroseptum and anterior wall segments.

On Day 4 of admission, the patient became acutely confused with expressive dysphasia. A cerebral CT scan demonstrated ischaemic infarcts in the right occipital and left temporal region with micro-haemorrhagic transformation. Repeat bedside echocardiography showed persistence of regional wall motion abnormalities. Additionally, a large pedunculated apical thrombus was identified. LMWH was changed over to unfractionated heparin and, eventually, to oral warfarin. His neurological deficit improved significantly (apart from residual mild expressive dysphasia), and the patient had complete recovery from his COVID-19 pneumonia (confirmed with two negative rt-PCR SARS-CoV-2 tests) with improved respiratory function.

**Figure 1:**
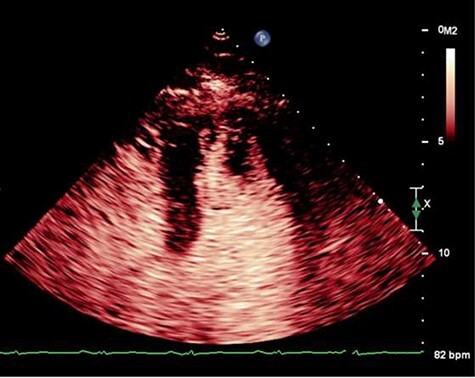
Pre-operative echocardiogram showing LV thrombus.

On Day 13, he developed acute global aphasia. Contrast echocardiography confirmed persistence of the same LV thrombus which had indeed increased in size despite 1 week of oral anticoagulation (Fig. 1). Given concerns for risk of haemorrhagic transformation of a recent ischaemic stroke with thrombolysis, a multi-sdisciplinary decision was taken to proceed to surgical thrombectomy.

**Figure 2:**
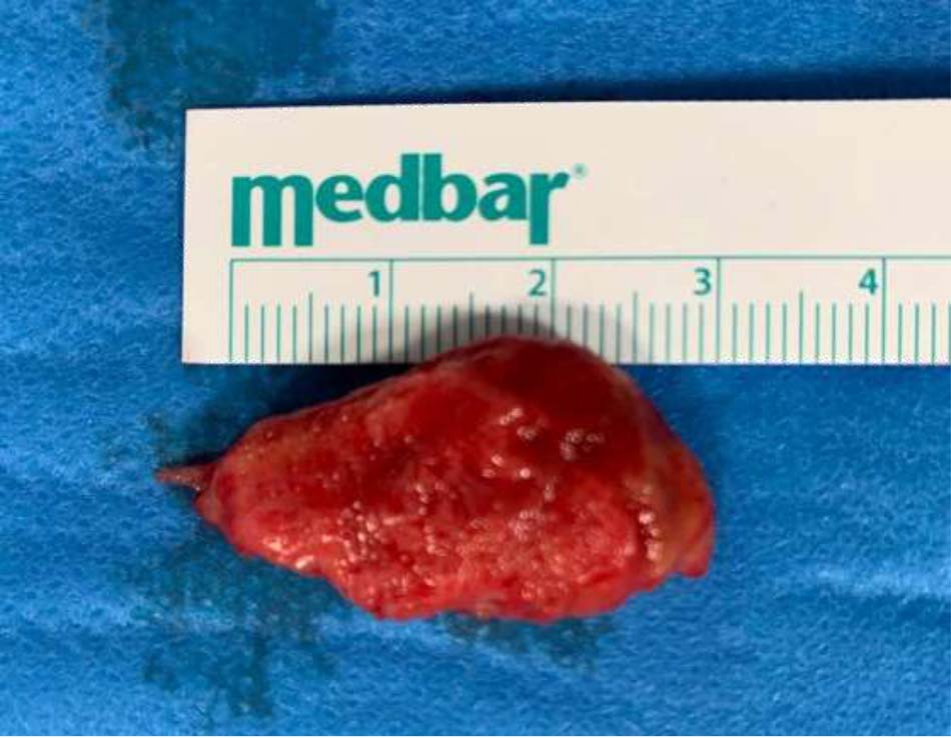
Thrombus folllowing extraction from left ventricle.

After median sternotomy, cardiopulmonary bypass with bicaval cannulation and LV venting was established by means of a right superior pulmonary vein vent. The bicaval cannulation approach was adopted to allow access to the thrombus via the transmitral approach, in case the aortic approach turned out to be unsuccessful. One litre of antegrade cold blood cardioplegia was delivered to arrest the heart, after which a transverse high aortotomy was performed. The thrombus was visualized and removed directly by means of a Magill forceps. The complete thrombus can be seen in Fig. 2. Transeoesophageal echocardiography confirmed the absence of any residual thrombus. Aortotomy was closed and the patient was weaned off cardiopulmonary bypass with no complications. Total cross-clamp time was 34 minutes with a bypass time of 68 minutes. His neurological function improved considerably and there was no limitation of his mobility. An MDT decision was made to keep the patient on oral anticoagulation for at least 1 year. Histopathological examination confirmed thrombus material and follow-up MRI confirmed complete resolution of thrombus.

## DISCUSSION

The formation of *in vivo* thrombus is dependent on Virchow’s triad, i.e. blood stasis, hypercoagulability and endothelial injury. Myocardial ischaemia (MI) is one potential mechanism behind endothelial injury and stasis. Regional wall abnormalities and formation of aneurysm can result in stasis, especially in anterior MI [3, 4]. In our case, the procoagulant state caused by SARS-CoV-2 infection [5] was superimposed on the endothelial inflammatory reaction and hypercoaguable state that occurs in the aftermath of an MI.

Pharmacological treatment is usually the first line of management for LV thrombus. In this case, thrombolysis was contraindicated given the patient had already had an ischaemic infarct with microhaemorrhagic transformation [6]. Surgery was preferred over further anti-coagulation and/or anti-platelet treatment given the mobility of the pedunculated thrombus and embolic phenomena that had already taken place despite adequate anticoagulation [7]. Hence, we report the first case of a LV thrombus associated with COVID-19 infection, treated successfully by an open surgical transaortic approach. While it is commonplace to approach such an aneurysm through a transapical ventriculotomy [8], we were concerned that LV myotomy might further compromise an already severely impaired LV. Although a transaortic approach was ultimately adopted, bi-caval cannulation was performed to allow conversion to a left atrial approach should there be difficulty in extracting a rather large thrombus through the aorta. As described by Tsukabe *et al.*, a Video-Assisted Thoracoscopic Surgery laparoscope was also kept on standby to aid visualization of the thrombus, but it was not required on this occasion [9].

While no significant difference has been demonstrated between pharmacological and surgical approaches, there is a tendency for fewer post-treatment thrombotic events in the surgical arm [10]. Surgical removal of LV mural thrombi via a transaortic approach may be considered in selected patients at high risk for recurrent embolism despite adequate anticoagulation.

## PATIENT CONSENT

The patient agrees that information and/or images, including intra-operative photography, could be used for medical education audit and research, including scientific publication. His name will not appear and he will not be identifiable in any way.

## DATA STATEMENT

Data sharing is not applicable to this article as no new data were created or analyzed in this study.
